# Editorial: Highlights of the 2nd D(dark grown)-root meeting

**DOI:** 10.3389/fpls.2023.1227490

**Published:** 2023-06-21

**Authors:** Katarzyna Retzer, Verena Ibl

**Affiliations:** ^1^ Department of Forest and Soil Sciences, Institute of Forest Ecology, University of Natural Resources and Life Sciences (BOKU), Vienna, Austria; ^2^ Laboratory of Hormonal Regulations in Plants, Institute of Experimental Botany, Czech Academy of Sciences, Prague, Czechia; ^3^ Department of Functional and Evolutionary Ecology, Molecular Systems Biology (MoSys), University of Vienna, Vienna, Austria

**Keywords:** droot, hidden half, dark grown root, root phenotyping, root growth adaptation

Environmental conditions have a strong impact on plant architecture and fitness (Pierik and Testerink, 2014). Roots are the below-ground organs of plants that sense and respond to environmental signals (Retzer and Weckwerth). Root growth, the root system architecture (RSA) but also overall plant performance is impaired by direct root illumination (Mo et al., 2015; Silva-Navas et al., 2015; Cabrera et al., 2022). Understanding how root illumination affects plant fitness is a growing research field. Therefore, the Research Topic was launched after the 2^nd^ D(dark grown)-root Meeting to present recent findings that decipher the impact of direct root illumination on plant performance. This Research Topic consists of seven articles by 31 authors (four original research articles, two review articles and one method paper), and includes studies on mono- and dicots.

RSA plasticity and root stress responses are crucial traits that underpin sustainability of plants that face changing environmental conditions (Retzer and Weckwerth). Roots adjust their growth direction when exposed to multiple stresses, such as light, gravity, salt or touch. Retzer and Weckwerth summarized known molecular mechanisms and diverse experimental setups that were applied. The flexibility of root growth adjustments depends on fine-tuned rearrangement of the actin cytoskeleton, which links environmental and hormonal signals to cellular responses (García-González and van Gelderen). Actin-binding proteins regulate the dynamic actin network assembly, but not much data is available on their impact on root growth, usually because of genetic redundancy (García-González et al., 2020). In their review, García-González and van Gelderen provide a comprehensive overview of the most current studies dealing with actin-binding proteins and actin cytoskeleton dynamics in roots responding to exogenous stimuli, including light triggered responses, and are highlighting outstanding gaps in the research field.

Plant development depends highly on light quality, direction, and intensity that drive photosynthesis in the shoot, which underpins the energy status and metabolic profiles of the plant (Retzer and Weckwerth, 2021). While shoots are directly exposed to light, roots of higher plants evolved to grow in the dark as described in the review by Retzer and Weckwerth.

Root and shoot communicate and distribute required resources among each other to achieve optimal growth depending on the sum of exogenous conditions (van Gelderen et al., 2018; Retzer and Weckwerth, 2021). To dissect how shoots and roots communicate with each other, two articles analyzed seedlings that were grown with roots shaded from direct illumination. Both studies showed that, on one hand, enhanced photosynthetic rate together with a well-established shoot-root communication result in most pronounced lateral root (LR) outgrowth, and that NRT2.1, a nitrate transporter, is a crucial player for shaping root architecture depending on resource availability (van Gelderen; Miotto et al.). Plants require not only sugars from the shoot to grow efficiently, but also diverse nutrients and water that are absorbed by the root from the soil (Retzer and Weckwerth 2021). Shade, which changes the light spectrum towards lower red but higher ratio of far-red wavelengths, delimits the photosynthetic rate in shoots. Furthermore, van Gelderen et al. show that depending on rich nitrate supplementation the root neglects shoot derived signals and continuous with growth. This effect is mediated by the transcription factor HY5 and the activity of NRT2.1.

Miotto et al. showed that the important role of NRT2.1 in root architecture modulation is depending not only on light quality, but also light quantity exposing the shoot. Low light intensity triggers signals from shoot to root, which strongly delimit LR development, whereby this response is lost in *nrt2.1* knock-out mutants (Miotto et al.). Together, both studies contribute to the understanding of the the central role of NRT2.1 in root growth regulation depending on overall resource availability. Moreover, according to the study of Miotto et al. it is important to point out that exogenous sucrose supplementation alone doesn’t compensate diminished low photosynthesis rates (Miotto et al.). Further studies are currently undertaken to decipher the complex molecular mechanisms that orchestrate shoot-root communication depending on balanced resource distribution between both organs (van Gelderen et al., 2023).

On the other hand, direct root illumination influences shoot performance too (Silva-Navas et al., 2015). As Paponov et al., reported, direct root illumination increased the accumulation of secondary metabolites in the shoot, which are substances that help plants deal with stress. Many secondary metabolites have medicinal properties and their production requires strict quality controls, therefore yield modulation by light in vertical farms, where plants can be grown under controlled conditions, is of agricultural interest. Popanov et al. introduce a device to illuminate the root specifically with LEDs emitting different wavelengths, and could measure clearly elevated levels of secondary metabolites in shoots of the medicinal plants, *Artemisia annua* and *Hypericum perforatum*.

Another possible commercial application of the research area is to understand how light affects adventitious root (AR) formation of stem cuttings. Stem cuttings are used by plant breeders to clone plants with desirable traits, but physiological and molecular details are not well described (Steffens and Rasmussen, 2016). Alallaq et al. dissected the impact of individual wavelengths on AR formation and the molecular mechanism that is involved in AR outgrowth of previously de-rooted Norway spruce seedlings. Illumination with red light, but not white light, impairs jasmonate (JA) and JA-isoleucine biosynthesis and repressing the accumulation of isopentyl-adenine-type cytokinins, which are compounds that repress AR initiation.

Finally, Dermenjiev et al. use an as near as possible to nature and sustainable approach to set-up a bench-top Dark-root device (DRD) BIBLOX (Brick Black Box) for distinct crops using 3D-printed rhizoboxes and LEGO® bricks for plant housing. The BIBLOX enables root growth as well as *in vivo in situ* early root tracking analysis in the dark of different crops in different soil conditions. This method paper further highlights the application of natural environmental conditions to controlled settings in the laboratory to improve the translation of the lab knowledge to the field again.

In conclusion, this first Research Topic on Dark-Grown-Root systems provides deeper insights into the effect of direct root illumination on overall plant productivity and fitness including novel technical improvements to cultivate plants using environmental parameters mirroring natural conditions.

## Author contributions

All authors listed have made a substantial, direct, and intellectual contribution to the work and approved it for publication.
